# Positive Psychological Capital Mediates the Association between Burnout and Nursing Performance Outcomes among Hospital Nurses

**DOI:** 10.3390/ijerph17165988

**Published:** 2020-08-18

**Authors:** Minjeong An, Eun Suk Shin, Myoung Yi Choi, Yeonhu Lee, Yoon Young Hwang, Miran Kim

**Affiliations:** 1 College of Nursing, Chonnam National University, 160 Baekseoro, Donggu, Gwangju 61469, Korea; anminjeong@jnu.ac.kr; 2 Department of Nursing, Chonnam National University Hospital, 160 Baekseoro, Donggu, Gwangju 61469, Korea; ses00296@hanmail.net (E.S.S.); sagijuju@naver.com (Y.L.); 3 Nursing Department, Chonnam National University Hwasun Hospital, 322 Seoyangro, Hwasun-eup, Hwasun-gun, Jeollanamdo 58128, Korea; choimy6@naver.com; 4 Seoul Women’s College of Nursing, 38 Ganhodaero, Seodaemungu, Seoul 03617, Korea; 5 Department of Nursing, Chunnam Techno University, 113 Daehakro, Okgwamyeon, Gokseong, Jeollanamdo 58128, Korea

**Keywords:** burnout, mediation, nurses, work performance

## Abstract

Nursing burnout is associated with reduced nursing performance outcomes. Positive psychological capital is known to play an important role in improving workers’ job performance. However, the association among the three variables has rarely been addressed. The purpose of this cross-sectional descriptive study was to explore the association between burnout and nursing performance outcomes among Korean nurses working at a tertiary hospital and the mediating role of psychological capital in this relationship. Recruited through convenience sampling, a total of 285 nurses provided data on their demographic characteristics and completed a structured questionnaire consisting of items from the Professional Quality of Life Scale (burnout), Nursing Performance Scale, and Psychology Capital Questionnaire. Descriptive statistics, student’s t-tests, one-way analysis of variance, Pearson’s correlation coefficients, and multiple linear regression analyses were used to analyze data. The significance of the mediation effect was obtained using a bootstrap approach with the PROCESS macro. The mean age of participants was 30.51 years, and most participants were females (94.0%) and unmarried (71.6%); more than half (57.5%) experienced a severe workload. The average (±standard deviation) scores of burnout, nursing performance outcomes, and positive psychological capital were 28.77 ± 4.93, 2.98 ± 0.32, and 3.19 ± 0.45, respectively. Burnout was associated with nursing performance among clinical nurses (β = −0.20, *p* < 0.001). Positive psychological capital mediated the association between burnout and nursing performance outcomes (β = 0.41, *p* < 0.001). These findings contribute to the understanding that burnout among nurses could be reduced by increased positive psychological capital, which results in improved performance outcomes. The findings also indicate that interventions to improve positive psychological capital should be developed and implemented for nurses’ burnout management and improvement in nursing performance outcomes.

## 1. Introduction

Nurses not only provide direct and indirect care to solve patients’ needs on the front line, but also maintain relationships with several hospital professionals and play diverse roles [1,2]. Recently, due to the rapidly changing clinical environment, such as the competitive environment of healthcare organizations, increasing demand for healthcare services for patients, and introduction of healthcare accreditation, high nursing performance outcomes are required from nurses [1]. Nursing performance outcomes mean the degree to which nurses achieve healthcare organizations’ goals by playing a role based on individual professionalism, and these indicate the actual execution of work related to all activities involved in providing nursing care [2,3]. In addition, good nursing work performance ensures that the patient is being provided with proper nursing care, and the improvement of performance ultimately translates to the improvement of nursing quality [1,4]. According to a previous study, nurses experience higher stress and more physical and psychosocial health problems compared with other professional groups; thus, the quality of nursing care and turnover is a concern [5]. Stress has a significant positive correlation with burnout, which decreases productivity and efficiency of nursing work and causes deterioration of nursing performance outcomes, impacting the ability to provide quality nursing care and causing loss to hospital management [3,6]. As a result, various efforts are being made to relieve the stress and burnout faced by nurses in their field and to improve nursing performance [7].

Social capital, trust, cooperation, and value sharing have been shown to contribute to the achievement of nursing organization goals by reducing organizational cynicism and turnover intention while increasing organizational commitment and job satisfaction [8,9]. In this regard, Luthans [10] referred to the development of personal positive organizational behavior to improve organizational performance and established the definition of positive psychological capital. Positive psychological capital is the concept of positive organizational behavior based on psychology emphasizing the importance of positive psychological values, and it is considered to produce more than traditional capital contributes to production [10]. Positive psychological capital awakens an individual’s existing maximum potential beyond human capital (i.e., individual’s knowledge, skills, and experience) and social capital (i.e., individual’s networks or relationships) [11]. It is composed of four independent components: self-efficacy, optimism, hope, and resilience [12]. Specifically, it cultivates the confidence needed to succeed in challenging tasks (self-efficacy), is optimistic about current and future success (optimism), and perseveres toward a positive motivational state, allowing individuals to have goal-directed energy and to be able to reestablish the pathways when needed to meet goals (hope). In addition, positive psychological capital provides a flexible capacity that, when faced with problems or challenges, deviates from them and returns to its original state or develops beyond it to attain success (resilience) [12]. These components, together or independently, can trigger personal motivation and influence efforts for success, leading to improvement in employee attitude, behavior, and work performance outcomes [13,14,15]. They can also help employees cope with contemporary work dynamics, be less affected by stressful or burnout events or situations [16,17], and immerse themselves in the organizations [13,14,16].

Nurses are one of the professional groups with very high risk of burnout [6,15]. Nurses who experience burnout feel negative emotions and attitudes toward patients [18] and fail to concentrate on work, causing patient safety-related problems [19,20]. In addition, physical and mental exhaustion due to burnout leads to a decrease in job-related achievement and satisfaction, causing nurses to consider turning over [15,21]. This atmosphere spreads to their colleagues, and adversely affects organizational performance [22]. Effective nursing workforce management is required to provide high-quality nursing care and improve nursing work performance; thus, it is essential to pay attention to nurses’ burnout prevention [18,23]. In this context, positive psychological capital having the four positive psychological values may play some role between burnout and performance outcomes in nurses. Previous studies identified the direct effects of positive psychological capital on burnout or nursing performance [4,15,22,24,25] and its mediating effect on nurses’ commitment to work [14] and on the performance of other workers [16]. However, there are insufficient studies examining the mediating role of positive psychological capital on burnout and performance outcomes of nurses.

Positive psychological capital is expected to contribute to the improvement of nursing quality and performance of nursing organizations through the improvement of positive psychology values of individual nurses [4,14]. Therefore, it is expected that it may have certain influence on the relationship between burnout and nursing performance. This study attempted to provide basic data for the future development of interventions and educational programs to improve nursing performance outcomes by confirming the role of positive psychological capital. The purpose of this study was to explore (1) the association between burnout and nursing performance outcomes and (2) the mediating effect of positive psychological capital on this association among nurses working at a tertiary hospital.

## 2. Materials and Methods

### 2.1. Study Design

This was a descriptive, correlational study.

### 2.2. Participants and Data Collection

In total, 285 nurses working at a tertiary hospital located in Gwangju City, South Korea, participated in this study. Sample size was calculated using the G*Power 3.1.9.4 program [26]. Based on multiple linear regression analysis with a significance level of 0.05, power of 90%, 10 explanatory variables, and small-medium effect size of 0.085, the minimum sample size required was 251 nurses. Accounting for a 20% sampling error, we planned to recruit 295 people; finally, 285 people participated in this study.

After obtaining approval from the institutional review board of the Chonnam National University (1040198–180202–HR–010–02, approved date: March 21, 2018), a tertiary hospital with more than 1,000 beds was conveniently selected, and nurses who had worked for at least 6 months were invited to participate. We obtained informed consent and collected data using a self-reported structured questionnaire from April to July 2018.

### 2.3. Measurements

Burnout was measured using 10 items that were part of the Korean version of Professional Quality of Life [27,28]. Each question was measured on a 5-point Likert scale, and the scores ranged from 10 to 50: 22 points or less was considered low, 23–41 points was moderate, and 42 points or more was high. McDonald’s ω coefficient was 0.78 in this study.

Nursing performance outcomes were measured using the Performance Measurement Scale [2], which included 4 dimensions: competency, attitude, willingness to improve, and application of nursing process. The scale consisted of 17 questions with a 4-point Likert scale (1, strongly disagree; 4, strongly agree), with higher scores indicating higher levels of nursing performance outcomes. McDonald’s ω coefficient was 0.93 in this study.

Positive psychological capital was measured using the Korean version of Psychological Capital Scale [16,29]. It consisted of 16 questions in 4 sub-areas: self-efficacy (6 questions), hope (4 questions), resilience (3 questions), and optimism (3 questions). Each question was rated on a 5-point Likert scale, and score ranged from 16 to 80, with higher scores indicating higher levels of positive psychological capital. McDonald’s ω coefficient was 0.92 in this study.

Demographic characteristics including age, sex, marital status, educational level, and perceived health status, as well as job-related characteristics including working department, working careers, and workload were assessed using the questionnaire.

### 2.4. Data Analysis

Data were analyzed using SPSS Statistics for Windows version 25.0 (IBM Corp., Armonk, NY, USA) and PROCESS macro v3.5 [30]. Descriptive statistics, including frequency, percentage (%), mean, and standard deviation (SD), were used to describe the sample, burnout, nursing performance outcomes, and positive psychological capital. Differences in nursing performance outcomes according to demographic characteristics and each characteristic variable were analyzed using student’s *t*-test and one-way analysis of variance test, and a post-hoc analysis was performed using the Bonferroni method. Pearson correlation was performed to examine the association between the subject’s burnout, positive psychological capital, and nursing performance. The mediating effect of positive psychological capital in the association between burnout and nursing performance outcomes was analyzed using Baron and Kenny’s three-step procedures [31] and Model 4 of the PROCESS macro [30]. In the first step, the effect of burnout on positive psychological capital was analyzed after controlling for demographic characteristics that showed differences in nursing performance. In the second step, the effect of burnout on nursing performance outcome was analyzed. In the third step, the effect of positive psychological capital on burnout and nursing performance was analyzed. Finally, the statistical significance of the mediating effect was confirmed using the bootstrap approach with the PROCESS macro [30]. The completely standardized indirect effect size was estimated by 95% bootstrap confidence interval (CI). The internal consistency of the scale was examined by calculating McDonald’s omega (ω) coefficient [31] because it required fewer and more realistic assumptions than alpha, especially for the measures with multidimensionality [32]. The statistical significance level was set to *p* < 0.05.

## 3. Results

### 3.1. Demographic and Job-Related Characteristics of Participants

The mean age of the participants was 30.51 ± 5.35 (mean ± SD) years, and 154 (54.0%) were less than 30 years old. Most of them were females (*n* = 268, 94.0%), unmarried (*n* = 204, 71.6%), and held a bachelor’s degree (*n* = 237, 83.2%). Approximately one-third of the participants were working at intensive care units (*n* = 97, 35.2%) and had been working for 3–7 years (*n* = 112, 39.3%). More than half of the participants evaluated their workload as high (*n* = 164, 57.5%), and 153 (53.9%) perceived their health status as relatively good (Table 1).

### 3.2. Differences in Nursing Performance Outcomes According to Demographic and Job-Related Characteristics

There were statistically significant differences in nursing performance outcomes according to age (F = 22.88, *p* <0.001), marital status (t = −4.33, *p* < 0.001), education level (F = 12.81, *p* < 0.001), working department, (F = 3.22, *p* = 0.023), and working career duration (F = 25.41, *p* < 0.001), but no significant difference was found according to sex, work intensity, and perceived health status. After performing a post-hoc test using the Bonferroni correction, results showed that nursing performance outcomes were significantly higher in older participants (30–49 years old) than in younger ones (23–29 years old). In addition, participants with a master’s degree had higher performance outcomes, compared with those with a bachelor’s degree or an associate degree. Regarding working career duration, nursing performance outcomes were significantly higher in participants working for ≥ 8 years, followed by 3–7 years, and < 3 years of work experience. In the post-hoc analysis, no significant difference was found in nursing work performance between working departments.

### 3.3. Burnout, Nursing Performance Outcomes, and Positive Psychological Capital, and Their Associations

The mean burnout score of nurses was 28.77 points (SD = 4.93). According to the burnout level categories, 33 participants had low burnout levels (11.6%), 250 had moderate levels (87.7%), and 2 had severe levels (0.7%) of burnout. The mean positive psychological capital score was 3.19 (SD = 0.45). Specifically, hope was the highest scoring sub-area with 3.33 points (SD = 0.52), followed by optimism with 3.22 points (SD = 0.59), resilience with 3.16 points (SD = 0.54), and self-efficacy with 3.10 points (SD = 0.52). The mean nursing performance outcomes score was 2.98 (SD = 0.32). Competency was the highest scoring dimension with 3.02 points (SD = 0.37), followed by application of nursing process with 2.99 points (SD = 0.38), attitude with 2.98 points (SD = 0.36), and willingness to improve with 2.91 points (SD = 0.41) (Table 2).

Nursing performance outcomes were negatively associated with burnout (r = −0.23, *p* < 0.001) and positively associated with positive psychological capital (r = 0.50, *p* < 0.001). Burnout was negatively associated with positive psychological capital (r = −0.50, *p* < 0.001).

### 3.4. Mediating Effect of Positive Psychological Capital between Burnout and Nursing Performance Outcomes

**Figure 1:**
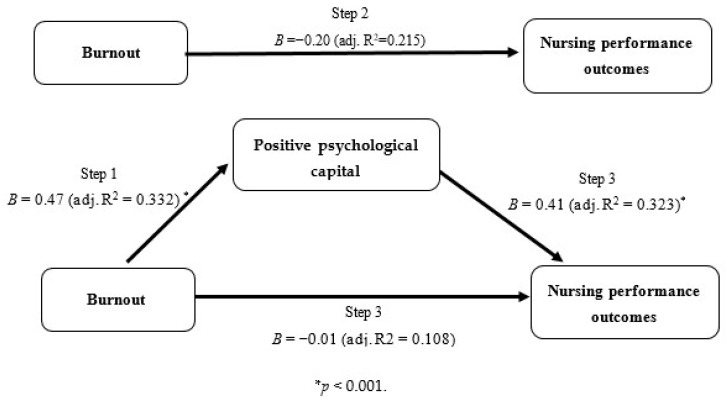
Mediating effect of positive psychological capital on the relationship between burnout and nursing performance outcomes.

Table 3 provides the results of multiple regression analysis performed to identify the mediating effect of positive psychological capital on the relationship between burnout and nursing performance outcomes. Table 4 presents the direct, indirect, and total effects. In step 1 (model 1), controlling for age, marital status, education level, and working career, burnout was significantly associated with positive psychological capital (β = −0.47, *p* < 0.001). In step 2 (model 2), burnout had a significant relationship with nursing performance outcomes (β = −0.20, *p* < 0.001). In step 3 (model 3), when both burnout and positive psychological capital were included in the multiple regression analysis model, only positive psychological capital was significantly associated with nursing performance outcomes (β = 0.41, *p* < 0.001), and the regression coefficient (β) for burnout decreased from −0.20 to −0.01. As burnout does not affect nursing performance outcomes, positive psychological capital was found to be a mediator of the relationship between burnout and nursing performance. The final model in step 3 showed 32.3% of the total variance of nursing performance outcomes of clinical nurses (Figure 1). The results of the non-parametric bootstrapping method confirmed the significance of the indirect effect of burnout through positive psychological capital (b = −0.01, 95% bootstrap CI = −0.02, −0.01). The completely standardized indirect effect size was −0.19 (95% bootstrap CI = −0.26, −0.12).

## 4. Discussion

This study examined the association between burnout and nursing performance outcomes, and further examined the mediating effect of positive psychological capital on this association among nurses working at a tertiary hospital. Our findings demonstrated that burnout was associated with nursing performance, and positive psychological capital played a mediating role when included in the association. The findings indicate that nurses with higher positive psychological capital may be less affected in burnout situations and have better nursing performance outcomes than their counterparts. In addition, they suggest that interventions and/or strategies increasing positive psychological capital may be helpful to improve nursing performance outcomes of nurses working in a tertiary hospital. Hospital administrators and health policy makers need to recognize the importance of positive psychological capital in nursing, and they should consider developing and providing interventions to promote better positive psychological capital among nurses with high risk of burnout.

Our findings showed that 88.4% of nurses experienced moderate to high burnout. This result is similar to the high levels of burnout (95.3–97.9%) found in previous studies conducted on Korean nurses [33,34]. In addition, our result was similar to the burnout levels of 73.1% to 86.9% reported by oncology nurses in China [35] and by intensive care unit nurses in Argentina [36]. As burnout has been reported to be significantly associated with mental problems such as depression and job dissatisfaction [21,37], active strategies are required to accurately assess nurses’ burnout. In particular, organizational and interpersonal resources have been shown to play an important role in reducing exhaustion in clinical nurses [21]. Thus, it is necessary to devise ways to efficiently utilize such resources to lower burnout.

In this study, burnout was identified as a significant predictor of nursing performance outcomes. This was in accordance with results indicating that the higher the burnout, the lower the nursing performance outcomes [18]. In addition, according to previous studies, burnout was identified as a potential inhibitor of nursing performance outcomes due to lower self-efficacy [38] and organizational commitment [15]. Therefore, managing burnout is important not only for improving the efficiency of nursing professional organization, but also for improving nursing performance outcomes, which ultimately has a significant impact on quality and outcomes of patient care [18].

This study found that positive psychological capital was significantly associated with nursing performance outcomes. Our findings are consistent with those of previous studies that reported the association of high positive psychological capital with high performance outcomes of nurses and other workers [4,16,24]. This indicates that positive psychological capital is a significant positive predictor for improving nursing performance outcomes. In other words, nurses who are confident, optimistic, hopeful, and resilient may attain better performance outcomes, as nurses with high levels of positive psychological capital do not easily give up or get frustrated, but are more likely to persist in terms of work engagement, especially when faced with problems or conflicts [16,17]. There are two ways through which hospital administrators can obtain and retain nurses with high levels of positive psychological capital. One is by hiring nurses with high positive psychological capital, and the other is to provide training programs to increase positive psychological capital among nurses, specifically through designing programs to facilitate an increase in each of the four psychological components (i.e., self-efficacy, optimism, hope, and resilience) [12,16].

Our findings showed that positive psychological capital had a mediating effect on the association between burnout and nursing performance outcomes. This finding was similar to the results of Luthans et al. [16], which reported that positive psychological capital had a mediating effect on the relationship between a supportive organizational environment and employee performance. Peng et al. [15] found that nurses with high positive psychological capital naturally experienced lower levels of burnout, because positive psychological capital consists of positive personal resources such as hope, self-efficacy, optimism, and resilience. This effect was identical in the United States, where newly graduate nurses with high positive psychological capital had significantly lower burnout and turnover intentions [25]. This result indicates that, despite burnout having a negative effect on nursing performance, it is possible to achieve desirable nursing performance outcomes by controlling this effect if the hospital nurse has adequate capacity in terms of positive psychological capital. Pan et al. [14] reported the impact of positive psychological capital on work engagement and suggested the need to strengthen such capital. Within these contexts, positive psychological capital may serve as a significant buffer against the impact of burnout on performance outcomes among hospital nurses. In addition, it may contribute in encouraging nurses to persist in their actions, resulting in good quality performance outcomes. Such capital is regarded as a state-like characteristic rather than a fixed-like trait, thus, it is possible to change the level of positive psychological capital through training and education [10,16]. Therefore, it is necessary to develop and apply an effective intervention program that can improve positive psychological capital among nurses to make it possible for them to manage their physical and mental distress, including stress and burnout. Further, more research should be conducted in this field on a variety of strategies and variables that can be used to develop positive psychology values.

In particular, our findings indicated that nursing performance outcomes were lower in nurses < 30 years old, unmarried, with a bachelor’s degree, and with less than 3 years of experience as a registered nurse. Thus, it is necessary to prioritize the development and application of positive psychological capital promotion programs for this specific group. In addition, short- to long-term nursing performance management plans need to be explored to continuously improve the levels of nursing performance outcomes according to changes in age, education, and working experience as a registered nurse. To our knowledge, this is the first study to identify the mediating effect of positive psychological capital in the relationship between burnout and nursing performance outcomes. It is significant that our findings provided a basis for improving nursing performance outcomes with the mediating role of positive psychological capital. In the future, a multidimensional approach according to the level of the individual, career, and working sites is required to effectively develop nurses’ positive psychological capital. In addition, interest and appropriate support for environments other than the hospital working environment (e.g., home, childcare, academic, and support environment) should be implemented.

Although our study provided evidence of the mediating role of positive psychological capital for the association between burnout and nursing performance, it had some limitations. Firstly, we recruited nurses working in a tertiary hospital using convenience sampling. The majority of the participants were females. As country, hospital level, and sex characteristics were not sufficiently reflected in the sampling, there were limitations for the generalizability of the study findings to nurses working in different levels of hospitals, including community care centers. Further studies need to be conducted with a representative sample containing various countries and hospital levels, and an appropriate ratio of sex. Secondly, because positive psychological capital is a personal resource, it can be influenced by various demographic and environmental characteristics besides the working environment of a tertiary hospital; thus, the results need to be interpreted carefully. Lastly, the causality could not be established because of the cross-sectional study design. Prospective studies need to be conducted to confirm the findings obtained from this study.

## 5. Conclusions

The findings of the present study provide empirical evidence on the significant role of psychological capital, in that it has a direct effect on work performance and an indirect effect in mediating an impact of burnout on work performance. Our results suggest that, despite enduring moderate to severe levels of burnout, nursing performance outcomes are more likely to increase when nurses have higher levels of positive psychological capital. Therefore, to maintain and/or improve nursing performance outcomes, customized interventions and/or strategies focused on improvement of positive psychological capital should be developed and provided for nurses with different levels of burnout and nursing performance. In addition, future studies should examine the effects of interventions developed for nurses.

## Figures and Tables

**Table 1:** Differences in nursing performance outcomes according to demographic and job-related characteristics of participants (*n* = 285).

Variables	Categories	*n*	%	Nursing Performance Outcomes			95% CI	ES
				Mean ± SD	t/F	*p*		
Age (Years)	Total			2.98 ± 0.32				
	23–29 ^a^	154	54.0	2.88 ± 0.25	22.88	<0.001 (a < b, c)	−0.29, −0.011 **	0.140
	30–39 ^b^	109	38.3	3.08 ± 0.35			−0.52, −0.19 ***	
	40–49 ^c^	22	7.7	3.23 ± 0.31			−0.32, 0.02 ^‡^	
Sex	Male	17	6.0	2.99 ± 0.28	0.07	0.944	−0.15, 0.16	0.033
	Female	268	94.0	2.98 ± 0.33				
Marital Status	Unmarried	204	71.6	2.93 ± 0.28	−4.33	< 0.001	−0.29, −0.11	0.604
	Married	81	28.4	3.13 ± 0.38				
Educational Level	AD ^a^	26	9.1	2.96 ± 0.21	12.81	<0.001 (a, b < c)	−0.15, 0.16 **	0.083
	BSN ^b^	237	83.2	2.96 ± 0.31			−0.56, −0.13 ***	
	Graduate ^c^	22	7.7	3.30 ± 0.37			−0.51, −0.18 ^‡^	
Working Department	Medical ward ^a^	79	27.7	2.91 ± 0.27	3.22	0.023 ^*^	−0.25, 0.02 **	0.034
							−0.16 0.28 ***	
	Surgical ward ^b^	82	28.8	3.03 ± 0.39				
							−0.24, 0.02 ^†^	
	ER ^c^	18	6.3	2.85 ± 0.32			−0.05, 0.40 ^‡^	
	ICU ^d^	97	34.0	3.02 ± 0.29			−0.12, 0.14 ^§^	
	Missing	9	3.2				−0.39, 0.05 ^¥^	
Working Careers (Years)	<3 ^a^	83	29.1	2.81 ± 0.27	25.41	<0.001 (a < b < c)	−0.30, −0.09 **	0.153
	3–7 ^b^	112	39.3	3.00 ± 0.30			−0.43, −0.21 ***	
	≥8 ^c^	90	31.6	3.13 ± 0.32			−0.23, −0.03 ^‡^	
Workload	Severe	164	57.5	3.00 ± 0.33	0.89	0.373	−0.04, 0.11	0.125
	Moderate	121	42.5	2.96 ± 0.31				
Perceived Health Status	Very bad ^a^	7	2.5	2.89 ± 0.29	0.56	0.640	−0.41, 0.26 **	0.006
	Bad ^b^	121	42.6	2.97 ± 0.30			−0.44, 0.22 ***	
	Good ^c^	153	53.9	3.00 ± 0.34			−0.60, 0.58 ^†^	
	Very good ^d^	3	1.0	2.90 ± 0.38			−0.14, 0.07 ^‡^	
	Missing	1	0.4				−0.44, 0.57 ^§^	
							−0.40, 0.60 ^¥^	

AD, associate degree; BSN, Bachelor of Science in Nursing; CI, Confidence Interval; ER, Emergency Room; ICU, Intensive Care Unit; ES, effect size (Cohen’s d for *t*-test or partial eta^2^ for ANOVA); a, b, c, and d were used as groups for comparison of post-hoc test or 95% CI; * Using the Bonferroni correction on post–hoc test, no significant differences were found between the groups; 95% CI for mean differences: ** a–b, *** a–c, ^†^ a–d, ^‡^ b–c, ^§^ b–d, ^¥^ c–d.

**Table 2:** Burnout, nursing performance outcomes, and positive psychological capital of participants (*n* = 285).

Variables	Categories	*n*	%	Mean	SD	Range
Burnout	Total			28.77	4.93	10–45
	Low (≤22)	33	11.6			
	Moderate (22–41)	250	87.7			
	Severe (≥42)	2	0.7			
Nursing	Total			2.98	0.32	2.00–4.00
Performance	Performance competency			3.02	0.37	1.86–4.00
Outcomes	Performance attitude			2.98	0.36	1.50–4.00
	Willingness to improve performance			2.91	0.41	1.33–4.00
	Application of nursing process			2.99	0.38	2.00–4.00
Positive	Total			3.19	0.45	2.00–4.00
Psychological	Self-efficacy			3.10	0.52	2.00–4.67
Capital	Hope			3.33	0.52	2.00–5.00
	Resilience			3.16	0.54	1.67–4.67
	Optimism			3.22	0.59	1.33–4.67

**Table 3:** Mediating effect of positive psychological capital on the relationship between burnout and nursing performance outcomes (*n* = 285).

Variables		Step 1 (Model 1)				Step 2 (Model 2)				Step 3 (Model 3)			
		Burnout → Psychological Capital				Burnout → Nursing Performance Outcomes				Burnout, Psychological Capital → Nursing Performance Outcomes			
		b	SE	β	*p*	b	SE	β	*p*	b	SE	β	*p*
Constants		4.27	0.16		<0.001	3.15	0.12		<0.001	1.91	0.22		<0.001
Age (years)	30–39	0.08	0.07	0.08	0.282	0.10	0.06	0.15	0.080	0.07	0.05	0.11	0.150
	40–49	0.20	0.12	0.12	0.108	0.14	0.09	0.11	0.154	0.08	0.09	0.06	0.378
Marital Status	Married	0.06	0.07	0.06	0.404	0.02	0.05	0.03	0.648	0.07	0.05	0.01	0.878
Education	BSN	0.02	0.08	0.02	0.786	0.03	0.06	0.04	0.615	0.02	0.06	0.03	0.666
	Graduate	0.32	0.12	0.19	0.009	0.21	0.09	0.17	0.028	0.11	0.09	0.09	0.195
Working Career (Years)	3–7	0.09	0.06	0.10	0.119	0.16	0.05	0.25	0.001	0.14	0.04	0.20	0.002
	≥8	0.05	0.10	0.06	0.583	0.16	0.08	0.23	0.036	0.14	0.07	0.21	0.042
Burnout		−0.04	0.01	−0.47	<0.001	–0.01	<0.01	−0.20	<0.001	<−0.01	<0.01	−0.01	0.868
Positive Psychological Capital										0.29	0.04	0.41	<0.001
Effect Size of Positive Psychological Capital										−0.19 (−0.26, −0.12) *			
R^2^ (Adjusted R^2^)		0.351 (0.332)				0.237 (0.215)				0.345 (0.323)			
F (*p*)		18.68 (<0.001)				10.71 (<0.001)				16.07 (<0.001)			

b, unstandardized coefficient; β, standardized coefficient; SE, standard error; BSN, Bachelor of Science in Nursing. References: age, 23–29; marital status, unmarried; education, associate degree; working careers, <3 years; * 95% bootstrap confidence interval of completely standardized indirect effect size.

**Table 4:** Direct and indirect effects on nursing performance outcomes.

Variables	Direct Effect	Indirect Effect	Total Effect	LLCI	ULCI	t	*p*
Burnout	−0.001	−0.013	−0.014	−0.020	−0.006	−3.76	<0.001
Psychological capital	0.290			0.205	0.375	6.72	<0.001
